# The Role of Low Mineral Water Consumption in Reducing the Mineral Density of Bones and Teeth: A Narrative Review

**DOI:** 10.7759/cureus.49119

**Published:** 2023-11-20

**Authors:** Kamalapriya V, Rekha Mani, Vijay Venkatesh, Seetha Kunhikannan, Shyam Ganesh V

**Affiliations:** 1 Conservative Dentistry and Endodontics, SRM Kattankulathur Dental College and Hospital, Chennai, IND; 2 Endodontics, SRM Kattankulathur Dental College and Hospital, Chennai, IND; 3 Dentistry, ICON Clinical Research Organisation, Chennai, IND

**Keywords:** water, dental caries, bone mineral density, low mineral water, reverse osmosis

## Abstract

Low mineral water has gained increasing attention due to its potential health implications concerning bone mineral density (BMD) and dental health. Reverse osmosis (RO) systems to purify water are in use extensively, and these systems, in addition to removing impurities from water, also remove 92-99% of beneficial minerals like calcium, lead, fluoride, magnesium, and iron. These minerals are essential for maintaining optimal mineral density of teeth and bones, thereby preserving bone and teeth health. Most of these mineral components are physically larger than water molecules and are trapped by the semi-permeable membrane of RO filters when drinking water is filtered through it. The resultant water is of very poor mineral content, and studies have shown that this water, when consumed, can absorb minerals from the body and eliminate the same through urine. The combined synergistic effect of consumption of low mineral water along with minerals being excreted has been shown to cause demineralization of bones and teeth, increasing the risk of osteoporosis and dental caries. This review tries to address the ill effects of consuming low mineral water along with preventive strategies to overcome its much-concealed adverse effects.

## Introduction and background

Safe drinking water is vitally important for healthy living. Water that is meant for human consumption, in other words, potable water, needs to be pathogen-free, devoid of hazardous chemicals, tasty, colourless, and odourless, and therefore suitable for drinking. The traditional ways of water purification like boiling, distillation, and filtration have been replaced by advanced methods like nano-filtration and ultra-filtration, adsorption and ion exchange, chemical and ultraviolet disinfection, and reverse osmosis (RO) [1]. RO water purification technology has spread its tentacles worldwide in recent years. RO filters work by forcing water molecules across a thick RO membrane to separate the contaminants. All suspended particles, including organic waste, colloids, and bacterial and viral contaminants, are removed during the RO process, in addition to salts in the ionic state. Therefore, RO systems have been extensively used to improve the quality of the water for drinking and also guarantee a water supply in an emergency [2].

While some homes possess RO systems, others rely on commercial enterprises. Twenty-litre bottles of bottled water are commonly used by homes for drinking and cooking. People think drinking bottled or RO water is safe; however, research has shown that the RO membrane virtually eliminates the water's mineral content. In India, various kinds of membranes are utilized. Membranes made of cellulose acetate, polyamide, and composite are frequently used [3]. As the global market for RO devices is expanding, there is still a dearth of scientific data on the ill effects of drinking RO-purified water [4]. Therefore, the aim of this review is to assess the effects of consuming RO-purified low mineral water on bone and teeth health.

## Review

The working mechanism of RO filters

Through the use of an RO membrane, RO technology isolates the solvent, typically water, from the solution. External pressure is required to balance the water's osmotic pressure. The process is permeation rather than spontaneous infiltration. When an external pressure is applied that is less than the solution's natural osmotic pressure, the solvent moves from the diluted solution to the concentrated solution. This process will successfully separate the solute and eliminate pollutants such as salt, colloid, bacteria, viruses, organic waste, and others [2]. One of the world's most cutting-edge membrane separation technologies is RO. RO separation has a one-time desalination rate of over 95% and can remove organic materials with a molecular weight of more than 150; in addition, it also removes bacteria and viruses from water to produce aseptic water. The workflow of RO systems includes tap water, mechanical filters, activated carbon filters, softeners, intermediate water tanks, booster pumps, security filters, RO high-pressure pumps, ozone generators, and 0.2-micron fine filters [2].

RO technology eliminates most of the ions from the water, especially divalent cations and anions [5]. There is abundant proof that fluoride was significantly reduced by the use of water purification systems based on RO technology. The systems based on RO, according to a study by Prabhakar et al., exhibited the largest reduction in fluoride levels, with the majority of filters completely removing fluoride from the water samples [6]. The semipermeable membrane used in RO has been shown to eliminate 94-98% of the calcium and magnesium. The calcium content of drinking water is therefore less than 6 mg/L in nations that use RO systems for purifying water.

In other words, residential filters or RO systems used to treat drinking water virtually eliminate calcium content [7]. Since demineralized water only contains minimal amounts of dissolved minerals like calcium and magnesium, which are the primary causes of hardness, the water produced can be regarded as an extreme form of low-mineral or soft water that has not been remineralized [8]. A study conducted in 1995 revealed that the fluoride levels were less than 0.3 ppm in 78% of the bottled water sold in Iowa City. Only 10% of samples were within the recommended optimal range of 0.7 to 1.2 ppm fluoride or exceeded it [8]. The filtration efficacy of essential minerals by RO systems is described in Table 1.

**Table 1:** Average purification efficacy of reverse osmosis (RO) systems Adapted from [9].

Component	Efficacy (%)
Calcium	97
Fluoride	95
Magnesium	96
Phosphate	95
Sodium	94
Sulphate	94
Iron	95
Zinc	95
Nitrate	90

Effects of drinking RO water on teeth

The effects of drinking RO water on dental health are a subject of interest, particularly in relation to calcium (Ca), magnesium (Mg), and fluoride (F) concentrations. Drinking water with appropriate levels of Ca, Mg, and F creates an environment conducive to remineralization in oral tissues, promoting dental health [10]. A study conducted by Razvan et al. [10] suggests that drinking water with Ca and Mg levels below recommended standards may not adequately support tooth remineralization. Furthermore, studies have indicated that the presence of calcium in drinking water can decrease the development of dental caries, as measured by the decayed, missing, and filled tooth surfaces (DMF-S). According to one study, calcium-containing water increases remineralization and decreases demineralization in early caries lesions [11].

This effect may be due to the relationship between calcium and fluoride levels in dental plaque, where calcium can infiltrate and provide additional sites for fluoride binding. Notably, drinking water should contain a higher proportion of free calcium compared to saliva, as saliva contains various binding agents, such as bicarbonate, proteins, and phosphorus, all contributing to significant calcium binding. Supplying water with 0.75 mg/L of fluoride and 90 mg/L of calcium, which yields a free calcium concentration over four times higher than that in saliva, can significantly reduce DMF-S while maintaining moderate concentrations of both ions [11].

Fluoride deficiency has been linked to an increased risk of tooth decay and a reduction in the formation of molar crowns within the dentine, which is dose-dependent. Fluoride's influence on the odontoblastic process, associated with dentine production, is facilitated through its interaction with the tubular structure and the metabolic activity of dentine. According to a study by Arvin et al., 100 mg/L of calcium has a protective effect equivalent to 0.64 mg/L of fluoride. Alternatively, the concept of calcium fluorite (CaF2) saturation is used to assess the combined impact of calcium and fluoride. Reducing calcium levels from 120 mg/L to 33 mg/L may increase the incidence of caries by as much as 46% [5].

An investigation conducted among school children in China found that low mineral water was associated with a slightly higher prevalence and incidence of caries on pit and fissure surfaces but slightly lower rates for smooth surfaces. This was the first promising epidemiological study to establish that drinking low mineral water increases the risk of dental caries and hypoevolutism among children in mixed dentition. The study further reported stunting among children caused by RO water consumption in their growing age [12].

Effect of drinking RO water on bones

Bone modelling, the process responsible for shaping and maintaining bone structure in childhood and adolescence, heavily depends on essential nutrients like calcium (Ca). However, Ca serves multiple crucial physiological functions beyond bone mineralization. The body employs a sophisticated regulatory system involving various tissues to oversee both bone formation and resorption, along with the absorption of Ca in the intestines. This complex regulation system is necessary to keep serum Ca concentrations within a narrow range. The concentration of Ca in the bloodstream also influences the secretion of cholecalciferol, commonly known as vitamin D, by the kidneys. A surge in serum Ca levels often resulting from excessive bone resorption can trigger a reduction in cholecalciferol levels. Cholecalciferol is well-established for its positive effects on bone health. As a result, continuous use of very low mineral drinking water might speed up bone resorption since it reduces cholecalciferol secretion, aggravating calcium insufficiency [13-15].

Furthermore, bone mineral density (BMD) is a key indicator of bone health and susceptibility to fractures. Paradoxically, drinking water with extremely low mineral content, such as RO water, can substantially decrease overall mineral intake, potentially raising health concerns, including the risk of osteopenia. To investigate the long-term effects of consuming very low mineral drinking water on bone modelling in children, Huang et al. conducted a comprehensive retrospective cohort study spanning four years. While RO water was not the sole water source in the study, it was discovered that using water with very low mineral content significantly reduced children’s daily mineral intake [13].

This reduction in daily mineral intake had several noteworthy consequences. Due to a drop in daily magnesium intake and a decrease in the consumption of bicarbonate from water, serum magnesium (Mg) concentrations decreased. Additionally, the lack of calcium (Ca) in drinking water may make Ca deficiency worse. It is interesting to note that youngsters who drank water with very low mineral content had higher serum Ca concentrations. This rise revealed an antagonistic link between dietary calcium consumption and total daily calcium intake. Consequently, the study’s findings suggest that in cases of inadequate Ca intake through drinking water, the body’s regulatory mechanisms adapt to maintain serum Ca balance [13].

Intriguingly, a study conducted by Grobler et al. unveiled an association between higher BMD values and elevated fluoride levels in drinking water. This suggests that as children progress in age, the minimal fluoride content in their water supply might not suffice to support increased bone density, potentially resulting in declining bone density over time [16].

Other health effects of drinking low mineral water

Low mineral water can lead to several health effects beyond its impact on bone health. Water with low mineral content can disrupt the body’s ability to maintain balance, affecting both water and mineral metabolism. This disruption can result in increased excretion of critical intracellular and extracellular ions from body fluids, leading to imbalances, changes in body water levels, and alterations in the functional activity of hormones related to water management. This may manifest as enhanced diuresis or increased urine production. Notably, low mineral water significantly increases the body’s ability to excrete sodium, potassium, chloride, calcium, and magnesium ions, along with changes in body water volume and serum sodium concentrations (typically around a 20% increase, on average) [12].

Remineralization of RO water

To counteract the negative consequences of drinking low mineral water, the idea of remineralizing RO water has been proposed. Better hydration, greater nutritional absorption, healthy digestion, remineralization of teeth and bones, and a stronger immune system are the primary health benefits. Because RO water has a significantly lower mineral concentration than drinking water, detrimental effects on the flavour of the RO water are possible [17].

The most popular technique for remineralizing RO permeate involves the permeate's liquid-solid interaction with a medium based on calcium carbonate and the addition of CO2. This process requires the procurement of a solid medium, together with the related difficulties of transportation and resource extraction [18]. To increase water hardness and chemical stability, RO permeate remineralization is necessary to change the concentrations of calcium and magnesium carbonate/bicarbonate. Recently, a hybrid electrodialysis method using bipolar membranes and ion exchange resins was created for RO permeate remineralization. In the investigated remineralization process, RO permeate remineralization uses a portion of the cation exchange resin regenerant stream, which is rich in primarily divalent ions [19].

Another strategy is to employ CO2 injection in conjunction with lime slurry, although this causes turbidity in the water and necessitates producing lime slurry on-site in a particular tank. Sodium bicarbonate and calcium sulphate can also be added to achieve remineralization; however, this approach is constrained particularly by the low solubility of calcium sulphate. Utilizing calcium bicarbonate or calcium chloride is another strategy that could be used. Other methods of remineralization include adding a mineral filter to an RO system to remineralize RO water, an alkaline water pitcher with a filter that restores essential minerals to drinking water, and the addition of trace mineral drops [18].

## Conclusions

Although RO water purification systems provide the safest drinking water to the human community, they also pose several adverse effects on systemic and oral health. The natural ability of the tooth to remineralize is depreciated due to the chronic consumption of RO water, which leads to the deterioration of incipient carious lesions and accelerates the development of cavitated carious lesions. RO water consumption may also be related to a declining BMD with an increased risk of osteoporosis and fracture. This is indeed alarming and is of colossal significance due to the generalized nature and chronicity of the issue, which tremendously affects human well-being.

An alternative proficient technology with remineralization of the RO water in moderate levels to meet the required daily amount of each micro and macro-mineral must be adapted to reap the benefits of the drinking water and to prevent adverse effects and ailments caused by the aforementioned. Knowledge about the mineral content of drinking water is essential to both the public and healthcare professionals. Future research in this regard can help us to overcome the health hazards of consuming low mineral water as well as develop new technologies that can provide safe drinking water while retaining its minerals.
